# Antioxidant and Anti-Atherosclerosis Activities of Hydrolyzed Jellyfish Collagen and Its Conjugate with Black Jelly Mushroom Extract

**DOI:** 10.3390/foods13152463

**Published:** 2024-08-04

**Authors:** Thunwa Binlateh, Pilaiwanwadee Hutamekalin, Soottawat Benjakul, Lalita Chotphruethipong

**Affiliations:** 1 School of Pharmacy, Walailak University, Thasala, Nakhon Si Thammarat 80160, Thailand; thunwa.bi@wu.ac.th; 2 Division of Health and Applied Sciences, Faculty of Science, Prince of Songkla University, Hat Yai, Songkhla 90110, Thailand; pilaiwanwadee.h@psu.ac.th; 3 International Center of Excellence in Seafood Science and Innovation, Faculty of Agro-Industry, Prince of Songkla University, Hat Yai, Songkhla 90110, Thailand; soottawat.b@psu.ac.th; 4 Department of Food Science, Faculty of Science, Burapha University, Mueang Chonburi, Chonburi 20131, Thailand

**Keywords:** hydrolyzed collagen, jellyfish, black jelly mushroom, conjugate, atherosclerosis

## Abstract

Atherosclerosis, a noncommunicable disease caused by cholesterol plaque, can cause chronic diseases. The antiplatelet medicines used in its treatment can cause complications. Marine collagen peptides can be used as a natural atherosclerosis remedy. The present study investigated the preparation and characterization of hydrolyzed collagen (HC) from jellyfish and its conjugation with black jelly mushroom extract (BJME). Their cytotoxicity and ability to prevent cholesterol-induced endothelial cell injury were also examined. HC was prepared using Alcalase or papain hydrolysis (0.2–0.4 units/g of dry matter (DM)). Higher yield, degree of hydrolysis, and antioxidant activities (AAs) were found in the HC obtained from Alcalase, especially at 0.4 units/g DM (A-0.4), compared to other processes (*p* < 0.05). Thus, A-0.4 was further conjugated with BJME (1–4%, *w*/*w* of HC). The HC-2%BJME conjugate showed the highest surface hydrophobicity and AAs compared to other samples. The FTIR spectra and size distribution also confirmed the conjugation between HC and BJME. When EA.hy926 endothelial cells were treated with HC or HC-2%BJME (25–1000 µg/mL), HC-2%BJME had no cytotoxicity, whereas HC at 1000 µg/mL induced cytotoxicity (*p* < 0.05). Both samples also exhibited protective ability against cholesterol-induced apoptosis and VE-cadherin downregulation of cells. Therefore, HC and conjugate could be natural agents for preventing atherosclerosis.

## 1. Introduction

Atherosclerosis is an inflammatory condition that involves the accumulation of cholesterol-rich plaque in the walls of arteries [1]. This phenomenon is associated with cardiovascular diseases such as stroke, myocardial infarction, and aortic aneurysm—leading causes of death worldwide [1]. As regulators of vascular homeostasis, endothelial cells (ECs) play a vital role in governing the pathological processes of atherosclerotic plaque formation [2]. In healthy arteries, ECs function as a barrier to the selective bidirectional movement of solutes and cells between intraluminal and extraluminal environments [2]. Changes in the endothelial barrier potentiate atherosclerosis by facilitating the dysregulated exchange of macromolecules such as cholesterol and LDLs and the infiltration of inflammatory cells, resulting in the accumulation of molecules and cells in the tunica intima, presenting as early atherosclerosis [3,4].

The integrity of the endothelial barrier is generally regulated by vascular endothelial (VE)-cadherin, a major component of the endothelial adherent junction in controlling vascular permeability and leukocyte transmigration [5]. Several studies have found that a reduced expression level of VE-cadherin increases vascular permeability, thereby enhancing the dysregulated exchange of atherogenic molecules and leukocyte infiltration [6]. Moreover, VE-cadherin expression is related to plaque instability, the degree of arterial occlusion, and atherosclerotic clinical events [7].

Hydrolyzed collagen (HC), especially from marine sources, has been extensively utilized as a dietary supplement for health [8]. Previous studies revealed that marine-derived HC exerts antioxidant potential for the treatment of diseases associated with oxidative stress [9,10]. For instance, HC from extracted salmon skin can scavenge DPPH^•^ radicals, reduce intracellular ROS levels, and prevent oxidation-triggered DNA damage [11]. Peptides from the HC of Spanish mackerel can effectively decrease hydroxyl radicals and superoxide anions and inhibit lipid peroxidation [9]. Moreover, collagen peptide from Siberian sturgeon had a cytoprotective effect on human umbilical vein endothelial cells against H_2_O_2_-induced oxidative damage [12]. Jellyfish, another high-quality marine source of HC, have recently raised interest due to their unique physicochemical and functional properties [13]. Jellyfish (*L. smithii*) is an economical raw material in Thailand and has been used to produce collagen [14,15]. Commercial proteases have mostly been used to prepare HC from jellyfish, where the differences in the processes, types, and levels of enzymes, affected the biological activities of the resulting HC [16,17]. HC from jellyfish possesses antioxidant, anti-aging, anticoagulant, and immune regulation activities [13,18,19]. Nevertheless, these activities were much lower than in other active compounds, particularly phytochemical compounds from plants. Therefore, improvement of new peptides to have strong bioactivity, especially antioxidant and anti-atherosclerosis activities, is necessary.

Conjugation between HC and plant polyphenols is an effective means to modify the structure and augment the activity of HC by increasing several hydroxyl and hydrophobic groups [10]. Commonly, phenolic compounds can prevent oxidative damage by stabilizing lipid peroxidation [20]. They also exert an inhibitory effect on the oxidation of LDLs, contributing to the prevention of atherosclerosis [21]. Black jelly (*Auricularia auricula-judae*) mushroom (BJM) is one source of natural bioactive compounds with antioxidant and anti-hypercholesterolemia activities [22]. A previous study revealed that *Auricularia auricula* extract could decrease the level of total cholesterol and the atherosclerosis index [22]. Thus, BJM extract might be a promising compound for the prevention of atherosclerosis. Currently, information on the anti-atherosclerosis activity of the bioactive peptides of HC from jellyfish and its conjugate with plant extract is limited. Therefore, this is the first study to investigate the preparation and characterization of HC from jellyfish and its conjugate with BJM extract. Furthermore, the activity of both compounds in terms of preventing cholesterol-induced endothelial injury was examined.

## 2. Materials and Methods

### 2.1. Materials

Alum-salted jellyfish (*L. smithi*) were acquired from the Mahachai market, Samut Sakhon, Thailand. The sample was desalted by washing with tap water until the salt content of the washing water was zero, which was monitored using a salinometer (Master-S/Mill α, Atago Co., Ltd., Tokyo, Japan) [17]. The obtained jellyfish were cut into small pieces (1 × 1 cm^2^), placed in food containers, and stored at 4 °C until use (<3 days).

Black jelly mushroom (5 kg) was purchased from the Makro department store, Chonburi, Thailand. Firstly, the sample was dried in a hot-air oven (Memmert, BE 500, Schwabach, Germany) at 50 °C until the moisture content was less than 10% wb. The dried sample was ground using a high-speed blender (Panasonic, Model MX-898N, Berkshire, UK) and packed in zip-lock bags. The obtained powder was kept at −20 °C until use.

### 2.2. Chemicals

Papain and Alcalase were obtained from Siam Victory Chemicals Co, Ltd. (Bangkok, Thailand). DMEM and fetal bovine serum were obtained from Gibco-Invitrogen (Paisley, UK). The chemicals used for antioxidant assays, including 2,2-diphenyl-1-picrylhydrazyl (DPPH), 2,2′-azino-bis (3-ethylbenzothiazoline-6-sulfonic acid) diammonium salt (ABTS), and 6-hydroxy-2,5,7,8-tetramethylchroman-2-carboxylic acid (Trolox), were purchased from Sigma-Aldrich, Inc. (St. Louis, MO, USA). The EA.hy926 cells were gifted by ATCC (Bethesda, MD, USA). VE-cadherin was obtained from Cell Signaling Technology (Danvers, MA, USA).

### 2.3. Effect of Various Enzymatic Hydrolysis Processes on Yield, α-Amino Group Content, and Antioxidant Activities of Hydrolyzed Collagen (HC) from Jellyfish

#### 2.3.1. Protease Activity Assay

The activity of papain and Alcalase was assayed at pH 7.0 and 40 °C for 15 min and at pH 8.0 and 50 °C for 15 min, respectively. The enzyme (0.2 mL) was mixed with 0.65% casein (0.2 mL), distilled water (0.2 mL), and phosphate buffer (625 μL). The mixture was then incubated for 15 min under the optimum condition tested. Afterward, 0.2 mL of cold 50% (*w*/*v*) trichloroacetic acid was added to terminate the reaction, and the mixture was kept on ice for 1 h. The supernatant was collected after centrifugation at 5000× *g* at 4 °C for 10 min. The oligopeptide content in the supernatant was measured using the Lowry assay [23], where Tyrosine was used as a standard. One unit of activity was defined as the amount of papain or Alcalase that liberated 0.01 µmol of Tyrosine per min (µmol Try/min) [24].

#### 2.3.2. Preparation of HC by Various Enzymatic Hydrolysis Processes

Desalted jellyfish were added to 2 volumes of distilled water, followed by adjustment to pH 7.0 or 8.0 using 1.0 M NaOH or 1.0 M HCl [10]. The mixtures were subjected to hydrolysis using papain or Alcalase at different levels (0.2, 0.3, and 0.4 units/g DM). The condition used for Alcalase hydrolysis was pH 8.0 at 50 °C for 120 min, and the papain hydrolysis condition was pH 7.0 at 40 °C for 150 min [24], the conditions used for both enzymes that showed the highest degree of hydrolysis. Thereafter, the mixtures were heated at 90 °C for 15 min to stop proteolysis. The obtained mixtures were centrifuged at 9000× *g* and 4 °C for 20 min and the supernatants collected for lyophilization. The lyophilized HC samples prepared with papain at levels of 0.2, 0.3, and 0.4 units/g of DM were called P-0.2, P-0.3, and P-0.4, respectively. HC preparations with Alcalase at levels of 0.2, 0.3, and 0.4 units/g of DM were called A-0.2, A-0.3, and A-0.4, respectively. All samples were analyzed as follows. 

##### Yield

The extraction yield of all samples was calculated via the following equation:

(1) $$Yield\;\left(\%\right)=\left\lfloor \frac{dry\;weight\;of\;HC\;(g)}{dry\;weight\;of\;initial\;jellyfish\;(g)}\right\rfloor \times 100$$

##### The α-Amino Group Content (α-AGC)

The α-AGC of all samples was measured as described by Chotphruethipong, Binlateh, Hutamekalin, Sukketsiri, Aluko and Benjakul [24]. HC solution was added to 0.2 M phosphate buffer (pH 8.2) (2 mL), followed by the addition of 0.01% TNBS solution (0.1 mL). The solution was mixed thoroughly and incubated at 50 °C for 30 min in the dark. The reaction was stopped by adding 0.1 M sodium sulphite (2 mL). The mixtures were then cooled at 25 °C for 15 min. The absorbance was read at 420 nm using a UV-1601 spectrophotometer (Shimadzu, Kyoto, Japan), and the α-AGC was reported as mmol Gly/g dry HC

##### Antioxidant Activities (AAs)

The DPPH and ABTS radical scavenging activities (DPPH-RSA and ABTS-RSA) were assessed as detailed by Wu et al. [25] and Arnao et al. [26] respectively. For DPPH-RSA, the samples (1.5 mL) were mixed with 0.15 mM DPPH in 95% ethanol (1.5 mL). The mixtures were incubated at 25 °C in the dark for 30 min. The absorbance of the solution was measured at 517 nm. Trolox (10–60 μM) was used as a standard. For ABTS-RSA, the samples (150 μL) were mixed with 2850 μL of ABTS solution and the mixtures were left at 25 °C for 2 h in the dark. The absorbance was then measured at 734 nm. The ferric reducing antioxidant power (FRAP) was examined [27]. The samples (150 μL) were mixed with FRAP solution (2850 µL) containing 25 mL of acetate buffer (300 mM; pH 3.6), 2.5 mL of 10 mM TPTZ solution, and 2.5 mL of 20 mM FeCl_3_·6H_2_O solution and kept for 30 min in the dark. The ferrous tripyridyltriazine complex was measured at 593 nm. Trolox (100–600 μM) was used as a standard for the ABTS-RSA and FRAP assays. The activities, including DPPH-RSA, ABTS-RSA, and FRAP, were expressed as µmol Trolox equivalents (TE)/g dry HC. Additionally, the metal chelating activity (MCA) was determined [27]. The samples (940 μL) were mixed with FeCl_2_ (2 mM; 20 μL) and ferrozine (5 mM; 40 μL). The mixture was allowed to stand for 20 min at 25 °C. The absorbance was then read at 562 nm. EDTA (10–60 μM) was used as a standard and the activity was expressed as µmol EDTA equivalents (EE)/g dry HC. 

The HC rendering the highest yield, α-AGC, and AAs was chosen to further determine the amino acid composition and conjugate preparation.

##### Amino Acid Composition 

The selected HC was first hydrolyzed by 4 M methanesulphonic acid containing 0.2% (*v*/*v*) 3-2 (2-aminoethyl) indole at 115 °C for 24 h and neutralized with 3.5 M NaOH. The digest was added into 0.2 M citrate buffer (pH 2.2). An aliquot (0.4 mL) was analyzed using an amino acid analyzer (MLC-703; Atto Co., Tokyo, Japan) [8]. The content of amino acid was reported as residues/1000 residues.

### 2.4. Preparation of Black Jelly Mushroom Extract (BJME)

Black jelly mushroom powders were mixed with distilled water at a ratio of 1:20 (*w*/*v*). Thereafter, the mixture was stirred using a shaker for 1 h at ambient temperature. This condition was obtained from a preliminary study in which it showed the highest total phenolic content. After extraction, the extracts were centrifuged at 10,000× *g* (CR22N, Hitachi, Hitachi Koki Co., Ltd., Tokyo, Japan) for 30 min at 25 °C. The supernatants were collected and filtered. The filtrates were freeze-dried, and the phenolic composition of the resulting extract was identified using liquid chromatography (LC)–quadrupole time-of-flight–MS (LC-QTOF-MS; 1290 Infinity II LC-6545 Q-TOF, Agilent Technologies, Santa Clara, CA, USA) [28].

### 2.5. Preparation of HC-BJME Conjugate

The HC-BJME conjugate was prepared using the free-radical grafting method [27]. Ascorbic acid (0.15 g) was mixed with 5 M H_2_O_2_ (0.5 mL) and then added into HC solution (1% *w*/*v*), followed by stirring for 2 h at 25 ± 2 °C. The resulting mixture was referred to as oxidized HC (OHC). The BJMEs (obtained from a previous study) at 1%, 2%, 3%, and 4% (based on HC weight) were subsequently added into the OHC solution and stirred at 4 °C for 24 h. Unconjugated BJME was separated by means of dialysis (3 kDa MWCO) at 4 °C for 24 h [27]. The proportion of conjugation between HC and BJME was calculated using the following equation.

(2) $$Conjugation\;(\%)=\left\lfloor \frac{Content\;of\;total\;BJME-Content\;of\;unconjugated\;BJME}{Content\;of\;total\;BJME}\right\rfloor \times 100$$

The conjugation percentages of HC-BJME conjugates at 1%, 2%, 3%, and 4% were 74.44%, 86.02%, 90.56%, and 90.90%, respectively. All the conjugates were lyophilized and kept at −40 °C until analysis.

#### 2.5.1. Analyses

All samples were examined for their surface hydrophobicity, in which ANS was used as a probe [29], and AAs (mentioned above). The HC-BJME conjugate having the highest surface hydrophobicity and AAs was selected for further characterizations, including FTIR analysis and size exclusion chromatography compared to the HC.

##### Fourier Transform Infrared (FTIR) Spectroscopy

The FTIR spectra of the samples, including the HC and the selected HC-BJME conjugate, were obtained using an FTIR spectrometer (Model EQUINOX 55, Bruker, Ettlingen, Germany). 

##### Size Exclusion Chromatography

Both samples were subjected to gel filtration chromatography using a Sephadex G-25 GF column (GE Healthcare, Bio-Science AB, Uppsala, Sweden). The absorbance of all fractions was read at 280 nm. The molecular weight (MW) of the samples was calculated based on MW markers [10].

### 2.6. Impacts of HC and HC-BJME Conjugate on Cell Viability and Prevention of Cholesterol-Induced Endothelial Cell Injury

#### 2.6.1. Cell Culture

EA.hy926 cells were cultured in DMEM supplemented with 10% FBS, and 100 U/mL penicillin–streptomycin in a humidified atmosphere supplied with 5% CO_2_. The culture medium was replaced every 2 days.

#### 2.6.2. Cell Viability Measurement and Hoechst33342 Staining

After being exposed to the HC or conjugate (25–1000 µg/mL) for 24 h, the cells were incubated with 0.5 mg/mL of MTT for 3 h at 37 °C. Afterward, the MTT solution was discarded, and the formazan crystals were dissolved with 100 µL DMSO. The absorbance was read at 570 nm using a microplate reader [30]. The percentage of cell viability was calculated relative to the control (without HC or conjugate). For Hoechst33342 staining, the cells treated with HC or conjugate for 24 h were stained with 10 µg/mL of Hoechst solution for 30 min in the dark. Then, the staining solution was discarded, and the cells were washed twice with PBS. Fluorescence images were captured using an Olympus IX73 inverted microscope [28]. Additionally, cell proliferation was measured after treatment with HC or conjugate (25–100 µg/mL) in DMEM without FBS for 24 h. After incubation, cell proliferation was also calculated, and data were reported relative to the control. The samples tested without cytotoxicity were selected for the next studies. 

#### 2.6.3. Prevention of Cholesterol-Induced Endothelial Cell Injury

##### Effect of Cholesterol on Endothelial Injury Induction

To evaluate the impact of cholesterol on triggering endothelial injury, cells were treated with 25, 50, and 100 µM of cholesterol for 24 h. Subsequently, cell viability and cell death were determined using the MTT assay and Hoechst33342 staining, respectively.

##### Anti-Atherosclerotic Potential of HC and Conjugate

The cells were pre-incubated with HC or conjugate at 50 µg/mL for 2 h before cholesterol exposure at 50 µM for 24 h. Cell viability and cell death were then assessed by means of MTT assay and Hoechst33342 staining, respectively. Endothelial integrity was also evaluated by measuring the expression levels of VE-cadherin using Western blot analysis [31]. Briefly, all proteins were extracted using lysis buffer. Equal amounts of protein were subjected to SDS-PAGE, transferred to a PVDF membrane, and incubated with primary antibody against VE-cadherin, followed by secondary antibody incubation [31]. The signal of the target protein was developed using chemiluminescence substrate. The densitometry of protein bands was quantified using ImageJ software (version Fuji, NIH Image, Bethesda MD, USA) in relative normalization to β-actin.

### 2.7. Statistical Analysis

A completely randomized design was used for all trials. Data were run in triplicate, and an analysis of variance (ANOVA) was conducted for all results. Means were compared using Duncan’s multiple range test. The Statistical Package for Social Sciences (SPSS 11.0 for Windows, SPSS Inc., Chicago, IL, USA) was used for analysis.

## 3. Results

### 3.1. Effect of Hydrolyzed Collagen (HC) from Jellyfish Prepared Using Various Processes on Yield, α-Amino Group Content (α-AGC), and Antioxidant Activities

#### 3.1.1. Yield 

Enzymatic hydrolysis processes had different impacts on the yield of the resulting HC. The yield ranged from 16.05% to 31.05% (Table 1). Increasing the levels of enzyme augmented the yield of HC (*p* < 0.05), regardless of the enzyme type used. Nevertheless, no difference was found in the yield of HC hydrolyzed by Alcalase at the levels of 0.3 and 0.4 units (A-0.3 and A-0.4) (*p* > 0.05). The yield of HC digested by Alcalase was also higher than that of HC hydrolyzed by papain at all levels of enzyme used (*p* < 0.05), implying that Alcalase could break the peptide bonds of protein in jellyfish more effectively than papain hydrolysis. 

#### 3.1.2. α-AGC

The α-AGC levels of the HCs are presented in Table 1. HCs prepared using different hydrolysis processes showed different α-AGCs, which varied from 0.03 ± 0.00 to 0.30 ± 0.00 mmol Gly/g dry HC. A result similar to that for yield was found (Table 1), in which the α-AGC was enhanced in a dose-dependent fashion with the amount of enzyme used (*p* < 0.05). A more obvious increase in α-AGC was attained in HC digested by Alcalase than by papain (*p* < 0.05). The result demonstrated that Alcalase hydrolysis drastically increased the cleavage of peptides in HC. 

#### 3.1.3. Antioxidant Activities (AAs)

All HCs tested donated protons and electrons as indicated by the DPPH-RSA, ABTS-RSA, and FRAP assays (Table 1). They also chelated metal ions. Among the samples tested, lower AAs were found for the HC obtained from papain hydrolysis than for that obtained from Alcalase hydrolysis (*p* < 0.05) (Table 1), regardless of the level of enzyme used. This finding was related to the yield and α-AGC (*p* > 0.05) (Table 1), in which Alcalase hydrolysis produced peptides with higher AAs in the HC. When considering the level of enzyme used, HCs obtained from both Alcalase and papain hydrolyses had augmented AAs with an increasing level of enzyme used (*p* < 0.05). The highest AAs were observed for the A-0.4 sample in all antioxidant assays (*p* < 0.05). Nevertheless, no difference in ABTS-RSA between the A-0.3 and A-0.4 samples was observed (*p* > 0.05). Because the HC obtained from Alcalase hydrolysis at 0.4 unit (A-0.4) had the highest yield, α-amino group content, and antioxidant activities, it was chosen to further measure the amino acid composition.

### 3.2. Amino Acid Composition of the Selected HC 

The amino acid composition of the HC obtained from the A-0.4 process is shown in Table 2. Gly (334.89 residues/1000 residues) was the dominant amino acid in the HC. Ala (91.99 residues/1000 residues), Glu (84.73 residues/1000 residues), and Pro (70.74 residues/1000 residues) were also detected in high amounts. Imino acid (Pro and Hyp) was also found. Moreover, the HC had a high level of hydrophobic amino acids, accounting for approximately 59% of the total amino acids. 

### 3.3. Identification of the Compounds in BJME Using LC-QTOF-MS

**Figure 1:**
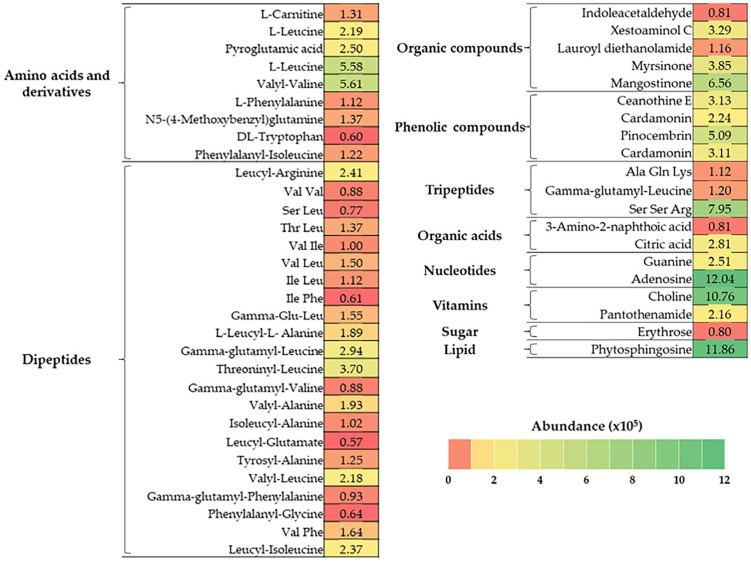
Heat map of bioactive compounds in BJME identified by LC-QTOF-MS. The color gradient represents the abundance (×10^5^) of the compounds.

As shown in Figure 1, BJME contained 10 groups of bioactive substances. Dipeptides (24.03%) and amino acids and derivatives (15.59%) were the major components found in BJME. Organic compounds (11.36%), nucleotides (10.55%), phenolic compounds (9.85%), and vitamins (9.37%) were detected in high abundance. Additionally, lipids (8.60%), tripeptides (7.45%), organic acids (2.62%), and sugars (0.58%) were found in BJME.

### 3.4. Characteristics of the HC-BJME Conjugate Prepared Using BJME at Different Levels

#### 3.4.1. Surface Hydrophobicity and Antioxidant Activities (AAs)

The surface hydrophobicity and AAs of HC, oxidized HC (OHC), and HC grafted with BJME at different levels (HC-BJME) are presented in Table 3. The oxidation process using hydroxyl radicals increased the surface hydrophobicity of HC (*p* < 0.05). The highest surface hydrophobicity was found for HC grafted with BJME at 2% (*p* < 0.05). At high levels of BJME (3% and 4%), a decrease in surface hydrophobicity of the conjugates was found (*p* < 0.05). Nevertheless, there was no difference in the surface hydrophobicity of both samples grafted with BJME at 3% and 4% (HC-3%BJME and HC-4%BJME) (*p* > 0.05). In general, surface hydrophobicity measures the change in the configuration of the proteins/peptides under harsh conditions, leading to structures unfolding and hydrophobic groups being exposed [32]. Thus, the increased surface hydrophobicity of HC indicated the formation of the conjugated peptides. However, adding BJME at high levels (>2%) decreased the surface hydrophobicity; it was noticed that the degree of surface hydrophobicity could be attributed to the level of BJME added.

When considering AAs (Table 3), a similar trend was found compared to surface hydrophobicity (Table 3). With BJME grafting, the HC-BJME conjugates’ AAs increased more than those of the HC or OHC (*p* < 0.05). The highest AAs of the conjugate were observed when the BJME level was increased to 2%. Nonetheless, levels of BJME higher than 2% decreased the AAs of the conjugates (*p* < 0.05), except MCA, in which the activity of the conjugates at 2% and 3% showed no difference (*p* > 0.05). Based on the results in Table 3, HC-2%BJME rendered the highest surface hydrophobicity and AAs; thus, it was selected for further characterization.

#### 3.4.2. FTIR Spectra

**Figure 2:**
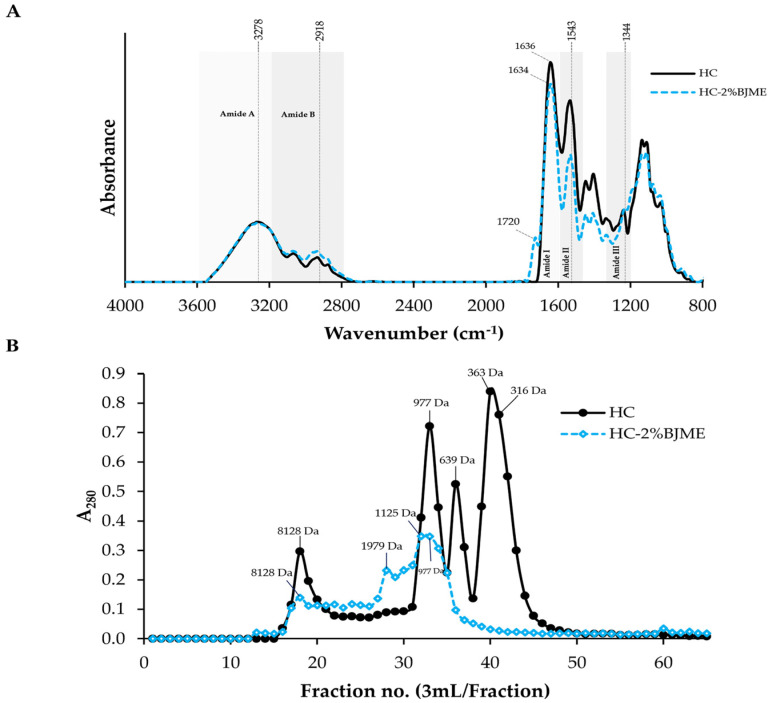
FTIR spectra in the wavenumber region of 4000–800 cm^−1^ (**A**) and elution profiles of HC and HC-2%BJME conjugate using SephadexTM G-25 gel filtration chromatography (**B**).

Figure 2A depicts the positions and wavenumbers of the FTIR spectral peaks of the HC and the HC-2%BJME samples. Both samples had similar peaks at ~3278 cm^−1^ (amide A, illustrating N-H stretching vibration) [33] and 2918 cm^−1^ (amide B, illustrating C-H stretching and NH_3_^+^) [24]. The positions of the amide II and amide III bands were found for both samples at wavenumbers of ~1543 cm^−1^ (N-H bending and C-N stretching vibrations) and ~1344 cm^−1^ (CH_3_ bending vibrations) [34], respectively. After conjugation with BJME, the new peak appeared at a wavenumber of ~1720 cm^−1^ (aromatic C=C) [35] for the HC-2%BJME sample, while it was not found in the HC sample (Figure 2A). Moreover, the position of the amide I band shifted to a lower wavenumber (from 1636 cm^−1^ to 1634 cm^−1^) in the HC-2%BJME sample. Apart from the aforementioned, a decrease in the intensity of amides I, II, and III was also found in the HC-2%BJME sample as compared to the HC sample. 

#### 3.4.3. Size Distribution

In general, the peptides with hydrophobic amino acids/phenolic compounds were detected at A_280_ [27,36]. The size of the peptides in HC ranged from 316 to 8128 Da (Figure 2B). With BJME grafting, new peaks with sizes of 1125 and 1979 Da appeared in the HC-2%BJME conjugate, while these peaks were not found in the HC sample. Additionally, the peaks for the low-molecular-weight peptides (<639 Da) of HC were absent in the HC-2%BJME conjugate, indicating that BJME could interact with HC and lead to a shift of the peptide peaks to a higher molecular weight.

### 3.5. Cytotoxicity of HC and HC-2%BJME Conjugate

**Figure 3:**
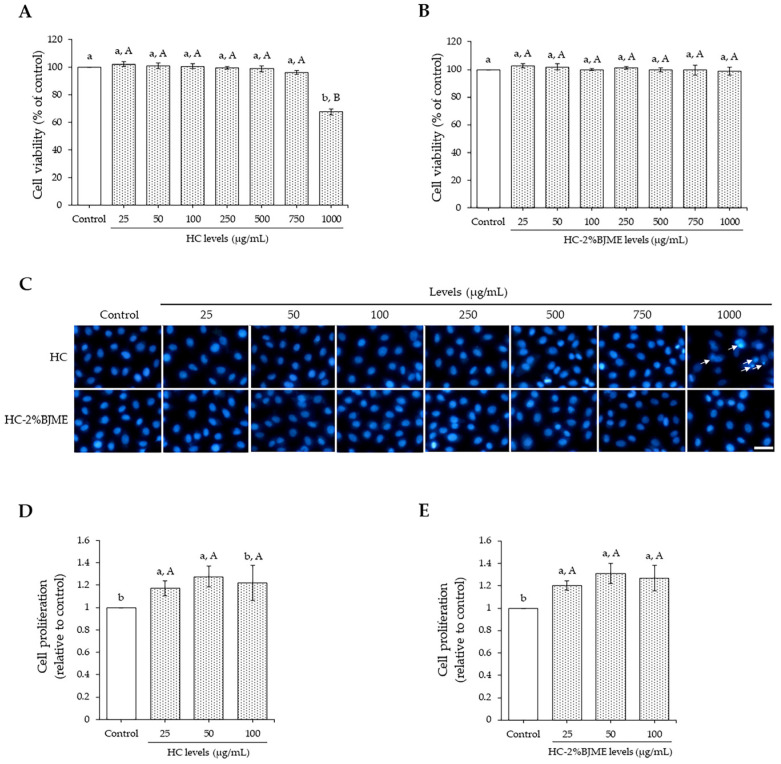
Endothelial EA.hy962 cell viability as affected by HC (**A**) and HC-2%BJME (**B**) at various levels. Nuclear morphology after treatment with HC or HC-2%BJME (arrows indicate apoptotic nuclear morphology; scale bar = 50 µm; magnification = 20×) (**C**) and cell proliferation of EA.hy962 cells as influenced by HC (**D**) and HC-2%BJME (**E**) treatments (25–100 µg/mL) for 24 h, respectively. Values are mean ± SD (*n = 3*). Different lowercase letters indicate significant differences among the samples tested (*p* < 0.05). Different uppercase letters indicate significant differences among the levels tested within the same sample group (*p* < 0.05).

As shown in Figure 3A,B, both HC and HC-2%BJME at 25–750 µg/mL did not affect endothelial EA.hy962 cell viability as compared to the control (*p* > 0.05). A high level of HC (1000 µg/mL) reduced cell viability (*p* < 0.05), while HC-2%BJME at the same level showed no cytotoxicity (*p* < 0.05). Hoechst33342 staining also confirmed the cell viability of both samples tested (Figure 3C), in which the HC at 1000 µg/mL could trigger cell death, as evidenced by the morphological features of apoptotic nuclei. Since the high levels (>100 µg/mL) and low levels (<100 µg/mL) of both samples did not differ in cell viability, the latter was chosen for the cell proliferation study. To investigate the effect of the selected samples on the proliferation of cells, FBS was removed from the culture medium. The results showed that samples at 25 and 50 µg/mL could increase endothelial cell proliferation, compared to the control (*p* < 0.05). Cell proliferation tended to decrease with the increasing level (100 µg/mL) of the samples (*p* > 0.05) (Figure 3D,E). Thus, the 50 µg/mL level of both samples was the proper level for subsequent studies.

### 3.6. Comparative Study of the Preventive Ability against Cholesterol-Induced Endothelial Injury of HC and HC-BJME Conjugate 

**Figure 4:**
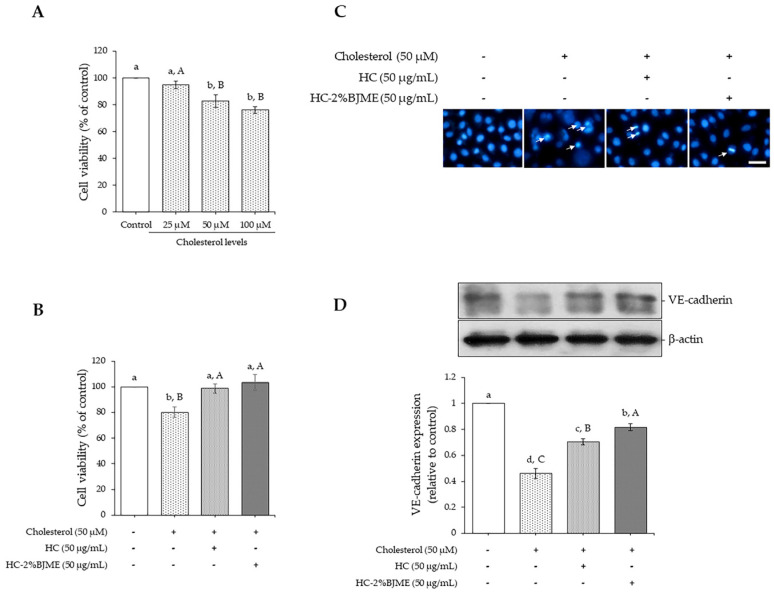
Effect of cholesterol (25–100 µM) on cell viability after incubation for 24 h (**A**). Impacts of HC and HC-2%BJME at 50 µg/mL on viability (**B**), nuclear morphology (arrows indicate apoptotic nuclear morphology; scale bar = 50 µm; magnification = 20×) (**C**) and VE-cadherin expression level (**D**) of cells induced by cholesterol at 50 µM. Values are mean ± SD (*n =* 3). Different lowercase letters indicate significant differences among the samples tested (*p* < 0.05). Different uppercase letters indicate significant differences among the levels tested within the same sample group (*p* < 0.05).

The potential of cholesterol to induce endothelial injury was initially evaluated. As depicted in Figure 4A, cholesterol caused a reduction in endothelial EA.hy962 cell viability in a dose-dependent manner (*p* < 0.05). Nevertheless, there was no difference in cell viability between cholesterol at 50 and 100 µM (*p* > 0.05). Thus, the 50 µM level was selected for the next study. We further determined the ability of HC and HC-2%BJME to prevent cholesterol-induced endothelial injury. The results showed that pre-treatment with HC or HC-2%BJME at 50 µg/mL prevented the decrease in cell viability induced by 50 µM cholesterol (*p* < 0.05) (Figure 4B). A slightly higher viability of cells was found in HC-2%BJME-pretreated cells compared to HC-pretreated cells (*p* > 0.05). Moreover, lowered apoptotic nuclei were observed for HC-2%BJME-pretreated cells compared to HC-pretreated cells (Figure 4C), indicating that HC-2%BJME could prevent the cell damage caused by cholesterol activation. To verify whether HC and HC-2%BJME prevented disruption of the endothelial barrier integrity induced by cholesterol, the integrity of the endothelial junction was thus evaluated. As illustrated in Figure 4D, the expression level of endothelial junctional protein VE-cadherin was significantly downregulated after cholesterol exposure, compared to control cells (*p* < 0.05). Conversely, pre-incubation with HC or HC-2%BJME could upregulate expression levels of VE-cadherin, compared to cholesterol-treated cells (without HC or HC-2%BJME). However, pretreatment with HC-2%BJME inhibited the decrease in the level of VE-cadherin expression further than pretreatment with HC (*p* < 0.05) (Figure 4D).

## 4. Discussion

This study used different enzymatic hydrolysis processes to produce hydrolyzed collagen (HC) from jellyfish. Alcalase hydrolysis provided a higher yield of HC than papain hydrolysis, especially when high levels of Alcalase were used (at 0.3–0.4 units) (Table 1). The treatment of jellyfish with Alcalase might have favored the digestion of proteins, resulting in the enhanced yield of HC. At high levels of enzymes, the protein distributed in jellyfish was hydrolyzed in a more pronounced manner than at low levels, leading to a higher yield. Nevertheless, the increased level of Alcalase at the higher 0.3 units did not increase the yield of HC. This was possibly due to saturation of the reaction rate [37].

When considering the α-AGC of HCs, the finding was consistent with the yield (Table 1), in which the α-AGC of HC obtained from Alcalase hydrolysis was higher than that derived from papain hydrolysis (Table 1). Alcalase preferentially cleaves peptides with hydrophobic residues, while papain prefers to hydrolyze peptide bonds having glycine or basic amino acids [24]. Protein substrates containing these hydrophobic peptides of jellyfish were likely hydrolyzed and released during the reaction at 50 °C for 120 min, resulting in the enhanced α-AGC. Chotphruethipong, Binlateh, Hutamekalin, Sukketsiri, Aluko and Benjakul [24] prepared HC from defatted seabass skin using Alcalase or papain hydrolysis, in which higher α-AGC was found in the HC obtained from Alcalase hydrolysis than that obtained from the papain hydrolysis counterpart. The use of Alcalase hydrolysis also provided a higher degree of hydrolysis (DH) than the use of papain for preparing HC from jellyfish (*Nemopilema nomurai)* [16]. However, the level of enzyme used influenced the DH. This study showed that increased levels of enzymes could augment the DH of the resulting HC in both processes implemented. 

Apart from the α-AGC of HCs, various proteolysis processes affected multiple antioxidant activities (AAs) (Table 1). This finding was related to the α-AGC, where higher AAs were found in the HC obtained from Alcalase hydrolysis than in that obtained from papain hydrolysis (*p* < 0.05). It was noted that Alcalase hydrolysis might produce peptides of HC from jellyfish with higher AAs. Consistent with Wang et al. [38], scallop protein hydrolysate prepared using Alcalase had higher DPPH-RSA and ABTS-RSA when compared to hydrolysates hydrolyzed using pepsin or dispase. As presented in Table 1, the increased level of enzymes contributed to the increased AAs. The A-0.4 sample exhibited higher AAs than the other samples for all assays (*p* < 0.05). A higher level of Alcalase hydrolysis might produce peptides with higher AAs than lower levels of Alcalase. Peptides containing aromatic and hydrophobic amino acids were reported to possess strong radical scavenging activity and electron donating ability [39,40,41]. In addition, peptides rich in Glx, Asx, Lys, His, and Arg chelate metal ions [42]. Thus, the HC had peptides that quenched both free radicals and metal ions.

When considering the amino acid composition of the A-0.4 sample (Table 2), the HC consisted of Gly at approximately 33.45% of the total amino acids, which was in line with the content of Gly found in edible jellyfish (30–33%) [43]. Similarly, Liu et al. [44] reported that Gly, Ala, and Glu were the dominant amino acids found in HC from *R. esculentum* jellyfish prepared using pepsin, followed by papain hydrolysis. Apart from these amino acids, the imino acid content of HC (85.3 mg/g) in the current study was lower than that of HC from *R. esculentum* jellyfish (101.8 mg/g) [44]. This difference in content is plausibly due to the difference in the species of raw material and process used. The amino acid composition of peptides is known to be a crucial factor in determining their biological activities [45]. Several studies have demonstrated that antioxidative peptides are generally short peptides with hydrophobic residues [8,46]. Thus, a high hydrophobic amino acid content (59%) in HC from jellyfish might increase its antioxidant activity.

When BJME was added to OHC (Table 3), the surface hydrophobicity of the HC-BJME conjugate increased compared to that of the HC (*p* < 0.05). The oxidation of HC using free radicals generated from H_2_O_2_ and ascorbic acid affected the structural changes in HC peptides, in which some internal hydrophobic domains were likely exposed more. The increased surface hydrophobicity of OHC confirmed this result. With BJME conjugation, the surface hydrophobicity of the HC-BJME conjugates was increased to the level of BJME, up to 2% (*p* < 0.05). The augmented surface hydrophobicity of the conjugates implied that BJME presumably bound to peptides via hydrophobic–hydrophobic interaction or hydrogen or covalent bondings leads to better characteristics of the resulting conjugate. Similarly, the HC-3%EGCG conjugate from seabass skin had higher surface hydrophobicity than native HC [10]. Nevertheless, the incorporation of BJME at levels above 2% reduced the surface hydrophobicity of the conjugates (*p* < 0.05). BJME comprises several bioactive compounds (Figure 1), which mostly have hydrophobic groups. An excessive level of BJME might increase polymerization between these compounds in BJME and the peptides of HC via hydrogen bonding or hydrophobic–hydrophobic interaction, leading to decreased surface hydrophobicity. Additionally, the compounds with high hydrophilicity, such as organic acids, organic compounds, and tripeptides (Figure 1), more likely augmented the hydrophilicity of conjugates and decreased their surface hydrophobicity [47]. Apart from surface hydrophobicity, the AAs of the conjugates were also determined (Table 3). The results were associated with the surface hydrophobicity (Table 3), in which HC-BJME conjugates showed higher AAs than did HC or OHC (*p* < 0.05) in all assays. Bioactive compounds, especially polyphenols with OH groups of BJME (Figure 1), might bind to peptides in HC via several bonds, resulting in enhanced AAs. Mangostinone, a xanthone derivative found in fungus, was documented to have powerful AAs [48,49]. Pinocembrin, a flavonoid identified in a variety of plants, possesses versatile AAs [50]. Myrsinone and cardamonin can scavenge ROS [51,52]. Therefore, grafting BJME, rich in polyphenols, with HC could increase the AAs of the conjugates. Song et al. [53] documented that the activities toward free radicals and the reducing ability of soy protein isolates (SPIs) grafted with young apple polyphenols were higher than those of SPI. Moreover, the BJME contained massive peptides with hydrophobic amino acids (Figure 1), which most likely increased the AAs of the conjugates [27]. However, HC had higher AA than OHC (*p* < 0.05). The loss of AA in OHC was probably due to the oxidation of peptides. When considering the levels of BJME grafted, the highest AA among the conjugates was observed when the level of BJME was increased to 2%. At high levels, the conjugates could interact with each other via hydrogen or hydrophobic interactions, decreasing the ability to provide protons/electrons and metal chelating, as evidenced by the lowered AAs of the conjugates with increasing levels of BJME above 2%**.** The HC from jellyfish had a high content of hydrophobic amino acids (59%) (Table 2), which likely interacted with the hydrophobic domains of polyphenols or hydrophobic peptides of BJME via a hydrophobic–hydrophobic interaction, resulting in lowered AAs. According to the surface hydrophobicity and AAs of the conjugates rendering the best result, HC-2%BJME was selected for further characterization. 

FTIR was used to determine the functional groups of the HC-2%BJME conjugate compared to HC (Figure 2A). Overall, there was no interaction between HC and BJME via the O-H group in the peptide backbone, as evidenced by no change in wavenumber at ~3240 cm^−1^ of both samples [52]. The conjugation of HC with BJME revealed the presence of a new peak at wavenumber ~1720 cm^−1^ (aromatic C=C) for the HC-2%BJME sample [35], indicating that phenolic compounds in BJME might bind to HC via H-bonding/hydrophobic–hydrophobic interaction, as shown by the presence of an aromatic peak for the conjugate. Additionally, lowering intensities of the amide I, II, and III peaks were found in the HC-2%BJME conjugate, compared to HC. Modification of the peptides by redox reactions might cause a change in the intensity/shift of HC peaks.

When considering the size distribution of both samples, the change in size of the peptides in the conjugate (Figure 2B) also confirmed that HC could conjugate with BJME, resulting in two new peaks of peptides formed with increased size (1125 and 1979 Da). Similarly, Yi et al. [54] revealed that an enhanced size of beta-lactoglobulin was detected when catechin was incorporated. Therefore, covalent conjugation between HC and BJME caused the change in the size of the HC peptides.

When HC or HC-2%BJME conjugate was added to EA.hy926 endothelial cells, the HC-2%BJME conjugate reduced the cytotoxicity more effectively than HC, as shown by the lack of cytotoxicity at a high level of the conjugate (1000 µg/mL) (Figure 3A–C). Consistent with the previous report, HC grafted with phenolic compounds not only increased the biological activities but also decreased the cytotoxic effect [55]. Based on the results in Figure 3D,E, both HC and HC-2%BJME conjugate at 50 µg/mL showed the highest cell proliferation, compared to other levels (*p* < 0.05). Thus, this level of both samples was selected for further investigation. Apart from the HC and HC-2%BJME levels, the level of cholesterol also influenced the cell viability, in which 50 µM was the maximum level needed to induce cell death (Figure 4A).

When EA.hy926 cells were acclimatized to evaluate the anti-atherosclerosis effect of HC and HC-2%BJME conjugate, cholesterol at 50 µM was used to induce cell death (Figure 4B,C) and VE-cadherin expression (Figure 4D). Pre-treatment with HC-2%BJME conjugate or HC at 50 µg/mL prevented the damage to cholesterol-activated cells, as indicated by the higher cell viability and VE-cadherin expression level than those of cholesterol-induced cells alone (*p* < 0.05). This was due to the presence of peptides with potent amino acids in HC (Table 2), which might exert various biological activities, particularly antioxidant and anti-platelet activities, leading to the prevention of endothelial cell injury and the regulation of inflammation processes related to oxidative stress [56]. When the ability of HC and HC-2%BJME to inhibit VE-cadherin downregulation was compared (Figure 4D), the latter showed higher activity than the former (*p* < 0.05). VE-cadherin is a major determinant of endothelial cell junction integrity, which regulates the permeability of blood vessels [5]. VE-cadherin expression at the endothelial cell-to-cell junction is downregulated in an atherosclerosis-susceptible region [57]. As a result, the vascular permeability and dysregulated exchange of atherogenic molecules are increased [57]. The higher expression level of VE-cadherin in HC-2%BJME-pretreated cells suggests that conjugation with BJME could increase the activity of HC in suppressing endothelial injury. BJME is rich in bioactive compounds, particularly adenosine, which is reported to have an endothelial-lowering effect by preventing endothelial barrier loss in oxidant-injured endothelial cells via the adenosine A1 receptor [58]. The presence of adenosine in BJME might increase the conjugating activity of HC-2%BJME. Thus, HC and its conjugate with BJME might serve as promising functional ingredients for the prevention of diseases related to atherosclerosis.

## 5. Conclusions

The preparation of hydrolyzed collagen (HC) from jellyfish using Alcalase at 0.4 units (A-0.4) can augment the yield, peptide cleavage, and antioxidant activities. Moreover, conjugation between HC and black jelly mushroom extract (BJME) yielded conjugated peptides with increasing surface hydrophobicity and antioxidant activities, especially when 2%BJME was incorporated. This was probably due to the bioactive compounds in BJME, in which the dipeptides, amino acids and derivatives, and organic compounds are massive substances. HC-2%BJME conjugate at 50 µg/mL increased the expression level of VE-cadherin in cholesterol-induced cells more effectively than HC. Further research is warranted to explore the potential application of HC or HC-2%BJME conjugate to alleviate atherosclerosis caused by cholesterol using both *in vitro* and *in vivo* models.

## Figures and Tables

**Table 1:** Yield, α-amino group content (α-AGC), and antioxidant activities of hydrolyzed collagen (HC) from jellyfish prepared using various enzymatic hydrolysis processes.

Samples	Yield (%)	α-AGC (mmol Gly/g dry HC)	Antioxidant Activities			
			DPPH-RSA (µmol TE/g dry HC)	ABTS-RSA (µmol TE/g dry HC)	FRAP (µmol TE/g dry HC)	MCA (µmol EE/g dry HC)
P-0.2	16.05 ± 0.49 ^e,C^	0.03 ± 0.00 ^f,C^	3.21 ± 0.09 ^d,B^	0.51 ± 0.06 ^c,B^	0.31 ± 0.06 ^c,A^	0.16 ± 0.01 ^d,C^
P-0.3	20.62 ± 1.14 ^d,B^	0.08 ± 0.00 ^e,B^	3.15 ± 0.05 ^d,B^	0.91 ± 0.02 ^b,A^	0.27 ± 0.03 ^c,A^	0.21 ± 0.01 ^c,B^
P-0.4	26.01 ± 0.15 ^c,A^	0.12 ± 0.00 ^d,A^	3.65 ± 0.08 ^c,A^	0.92 ± 0.07 ^b,A^	0.24 ± 0.00 ^c,A^	0.27 ± 0.01 ^b,A^
A-0.2	29.62 ± 0.66 ^b,B^	0.27 ± 0.00 ^c,C^	3.81 ± 0.04 ^b,B^	0.99 ± 0.00 ^b,B^	0.26 ± 0.01 ^c,C^	0.27 ± 0.00 ^b,B^
A-0.3	30.14 ± 0.58 ^ab,AB^	0.29 ± 0.00 ^b,B^	3.85 ± 0.12 ^b,B^	1.14 ± 0.00 ^a,A^	0.38 ± 0.06 ^b,B^	0.28 ± 0.00 ^b,B^
A-0.4	31.05 ± 0.57 ^a,A^	0.30 ± 0.00 ^a,A^	4.18 ± 0.05 ^a,A^	1.16 ± 0.00 ^a,A^	0.50 ± 0.02 ^a,A^	0.45 ± 0.00 ^a,A^

Values are mean ± SD (*n = 3*). Different lowercase letters in the same column indicate significant differences among all samples tested (*p* < 0.05). Different uppercase letters in the same column indicate significant differences within the same enzyme used (*p* < 0.05). P-0.2, P-0.3, and P-0.4: HC prepared from jellyfish using papain at 0.2, 0.3, and 0.4 units/g DM at 40 °C for 150 min, respectively; A-0.2, A-0.3, and A-0.4: HC prepared from jellyfish using Alcalase at 0.2, 0.3, and 0.4 units/g DM at 50 °C for 120 min, respectively.

**Table 2:** Amino acid composition of HC from jellyfish prepared using the A-0.4 process *.

Amino Acid	Content (Residues/1000 Residues)
Alanine (Ala)	91.99
Arginine (Arg)	55.95
Asparatic acid (Asp)	66.88
Cysteine (Cys)	2.71
Glutamic acid (Glu)	84.73
Glycine (Gly)	334.89
Histidine (His)	24.97
Isoleucine (Ile)	18.11
Leucine (Leu)	30.98
Lysine (Lys)	29.09
Hydroxylysine (Hylys)	34.19
Methionine (Met)	3.70
Phenylalanine (Phe)	6.40
Hydroxyproline (Hyp)	49.90
Proline (Pro)	70.74
Serine (Ser)	30.31
Threonine (Thr)	29.20
Tyrosine (Tyr)	4.93
Valine (Val)	29.84
Tryptophan (Trp)	0.48
Total	1000.00
Imino acid (Hyp + Pro)	120.64

* A-0.4 process (0.4 units of Alcalase).

**Table 3:** Surface hydrophobicity and antioxidant activities of hydrolyzed collagen (HC) from jellyfish, oxidized HC (OHC), and HC conjugated with black jelly mushroom extract (BJME) at different levels.

Samples	Surface Hydrophobicity	Antioxidant Activities			
		DPPH-RSA (µmol TE/g dry HC)	ABTS-RSA (µmol TE/g dry HC)	FRAP (µmol TE/g dry HC)	MCA (µmol EE/g dry HC)
HC	99.85 ± 4.25 ^e^	4.18 ± 0.05 ^d^	1.16 ± 0.01 ^d^	0.50 ± 0.02 ^e^	0.45 ± 0.00 ^c^
OHC	110.56 ± 6.90 ^d^	2.30 ± 0.02 ^e^	0.82 ± 0.02 ^e^	0.34 ± 0.00 ^f^	0.50 ± 0.04 ^c^
HC-1%BJME	130.15 ± 9.60 ^c^	9.07 ± 0.20 ^b^	3.44 ± 0.02 ^c^	1.39 ± 0.00 ^d^	2.78 ± 0.18 ^b^
HC-2%BJME	207.29 ± 1.62 ^a^	10.69 ± 0.14 ^a^	3.73 ± 0.01 ^a^	1.76 ± 0.02 ^a^	3.57 ± 0.15 ^a^
HC-3%BJME	194.23 ± 1.26 ^b^	8.15 ± 0.15 ^c^	3.50 ± 0.04 ^b^	1.51 ± 0.01 ^c^	3.54 ± 0.12 ^a^
HC-4%BJME	185.24 ± 5.91^b^	7.97 ± 0.07 ^c^	3.52 ± 0.03 ^b^	1.54 ± 0.02 ^b^	2.88 ± 0.21 ^b^

Values are mean ± SD (*n = 3*). Different lowercase letters in the same column indicate significant differences among all samples tested (*p* < 0.05).

## Data Availability

The original contributions presented in the study are included in the article, further inquiries can be directed to the corresponding author.

## Abbreviation

a-AGC	Alpha-amino group content.
AAs	Antioxidant activities
ABTS-RSA	ABTS radical scavenging activity
BJME	Black jelly mushroom extract
DH	Degree of hydrolysis
DM	Dry matter
DMEM	Dulbecco’s modified Eagle medium
DPPH-RSA	DPPH radical scavenging activity
EC	Endothelial cell
FBS	Fetal bovine serum
FRAP	Ferric reducing antioxidant power
FTIR	Fourier transform infrared spectroscopy
HC	Hydrolyzed collagen
LC-QTOF-MS	Liquid chromatography–quadrupole time-of-flight–mass spectrophotometry
LDLs	Low-density lipoproteins
MCA	Metal chelating activity
OH	Hydroxyl
SPI	Soy protein isolate
VE-cadherin	Vascular endothelial cadherin
